# Efficacy of Amniotic and Chorionic Membrane in Facial Wound Healing: A Comparative Study

**DOI:** 10.7759/cureus.58160

**Published:** 2024-04-12

**Authors:** Indra Kumar Periyasamy, Ayisha Mehthaf, Gayathri Priyadarshini Elangovan, Vijayalakshmi D, Gowthaman Vijaykumar, Ahila Elumalai

**Affiliations:** 1 Oral and Maxillofacial Surgery, Vivekanandha Dental college for women, Tiruchengode, IND; 2 Oral and Maxillofacial Surgery, Vivekanandha Dental College for Women, Tiruchengode, IND; 3 Periodontology, Vivekanandha Dental College for Women, Tiruchengonde, IND; 4 Oral Pathology, Dhanalakshmi Srinivasan Dental College, Perambalur, IND; 5 Dentistry, Ex-servicemen Contributory Health Scheme (ECHS) Polyclinic Ministry of Defence, Chennai, IND; 6 Periodontics, Sri Venkateshwaraa Dental College, Puducherry, IND

**Keywords:** dehydrated human amnion, healing rates, wound duration, placental grafts, asepsis, wound healing, wound size, facial wounds, chorion, amnion

## Abstract

Background

Advancements in regenerative techniques have been utilized in placental amnion and chorion for a variety of purposes. Their ability to regenerate tissues has led to their usage in tissue engineering, wound healing, and other therapeutic applications. This study aims to evaluate and compare the efficacy of amnion and chorion in facial tissue wound healing.

Methodology

The study was an observational comparative study conducted in the Department of Oral and Maxillofacial Surgery, involving 20 participants divided into two groups (Group I and Group II). Study groups were selected according to the inclusion and exclusion criteria. A dehydrated human amnion/ chorion membrane was applied to the affected site of each group respectively. Its efficacy in wound healing was analyzed in the first, third, seventh day, and second week. Statistical analysis was done using SPSS software (IBM Corp., Armonk, NY).

Results

Patients treated with amnion membrane showed a decrease in wound size and the wound was completely healed by second week with mean scores of wound sizes of 0.00 whereas the wound remained unhealed by second week with mean of 1.70 to those treated with chorion membrane.

Conclusion

Amnion showed superior efficacy in wound healing at two-week intervals when compared to the chorion. Hence, this could be used in regenerative medicine as a graft to induce healing in facial wounds.

## Introduction

The past few decades have witnessed an increase in the number of people becoming more concerned with their skin [1,2]. However, the wounds caused by domestic violence, assault, chemical burns, etc., lead to loss of integrity of the skin and are a major cause of morbidity and mortality. Facial injury treatment is of major concern for patient aesthetic acceptance [3]. The primary goals in treating wounds are rapid closure, restoration of function, and aesthetically satisfactory scar development [3,4].

The wound is the breakdown in the protective function of the skin; and loss of continuity of epithelium with or without loss of underlying connective tissue [5]. The wound healing process is a collection of well-coordinated processes of cell migration, proliferation, and extracellular matrix deposition undergoing three overlapping but distinct phases of inflammation, proliferation, and maturation [6,7]. However, an imbalance in any of these three phases may lead to harmony in the disruption of the normal wound healing process, resulting in the transformation towards chronic nonhealing wounds and abnormal scar formation [8].

Different materials have been used in the past in the field of surgery to promote healing [9]. Human amniotic membranes have been used for over 70 years. It was used for burned and ulcerated skin surfaces by Stern in the year 1913 [9]. The amnion membrane is the innermost layer of the placenta consisting of a thick basement membrane and avascular stromal matrix [10,11]. The chorion membrane is the outermost layer covering the placenta [10-13]. These membranes derived from the placenta are rich in cytokines and growth factors. They possess the potential ability to overcome the limitations of the present treatment procedures [12]. Because it has no immune response decreases scar tissue [14], serves as a biological barrier [14], reduces pain [15,16], inflammation increases the healing process [17], and also has an antimicrobial effect [18,19].

Owing to these properties it has been used in the field of ophthalmology and stem cell biology. Early use of fresh amniotic membranes containing both amnion and chorion membranes has proved beneficial in treating ulcers, burns, and dermal injuries [20]. While the basis for this healing effect has not been fully cleared, native human amnion/chorion membrane contains more amount of growth factors, including epidermal growth factor, basic fibroblast growth factor, keratinocyte growth factor, transforming growth factor, nerve growth factor hepatocyte growth factor that is known to play critical role in the physiological processes leading to normal wound healing and tissue regeneration [21]. Previous studies demonstrating the anti-inflammatory effects of amniotic membrane transplantation in ocular surface disorders in 2001 stated that inflammatory cells get trapped and undergo apoptosis in the matrix of the amniotic membrane [12,21]. Hao et al. demonstrated anti-angiogenic and anti-inflammatory proteins in the human amniotic membrane in 2000. They concluded that human amniotic membrane epithelial and mesenchymal cells express various anti-angiogenic and anti-inflammatory proteins and some of those proteins also were found in amniotic membrane stroma [22]. Ajbani et al. concluded the anti-inflammatory effect of chorion membrane in periodontal pocket therapy stating that chorion membrane has an anti-inflammatory effect [23]. The major objective of this study was to compare the efficacy of amniotic and chorionic membranes in facial wound healing.

## Materials and methods

Study design

This was an observational comparative study that was conducted in the Department of Oral and Maxillofacial Surgery in a Dental College in south India. It was conducted after getting approval from the institutional ethical committee (VDCW/IEC/153/2019).

Study participants

Patients are able and willing to provide consent and agree to comply with study procedures along with follow-up evaluation and showed no clinical signs of infection were included in the study. Patients having facial abrasions visiting the Department of Oral and Maxillofacial Surgery of the tertiary care hospital were only considered. Patients currently participating in another study were not considered. Patients receiving radiotherapy and chemotherapy or having noncommunicable systemic illnesses like diabetes mellitus or hypertension were excluded. Pregnant and lactating women were also not recruited.

The study group included a total of 20 participants which were divided into two separate groups group 1 with 10 participants and group 2 with 10. The study groups were selected according to the above-mentioned inclusion and exclusion criteria. In group 1 chorionic membrane was applied and in group 2 allantoic membrane was applied. 

Data sources

Twenty patients with abrasion wounds who met the inclusion criteria were selected for the study. All the participants were explained about the procedure in their understandable language and written informed consent was obtained. The chief complaint, etiological factor, and site of abrasion were recorded for every patient. All the participants were then randomly allocated into two groups. Equal distribution was done in both the groups, respectively, who received amniotic membrane treatment and chorion membrane.

Povidone iodine was used as an antiseptic to clean the wound. The first group of patients was placed with an Amniotic membrane and the other group was placed with a Chorion membrane. The membranes used were hydrated once placed over the abraded surface so they became adherent to the abraded surface. Once the membrane was placed it was covered with an elastic self-adhesive bandage for 24 hrs to secure the membrane over the abraded surface.

Patients were recalled on the first, third seventh, and 14th day to check for wound healing and asepsis score. The outer dressing was removed after 24 hrs and povidone iodine was used to remove any exudate present on the abraded surface. After that the following scores were recorded for all the patients the wound healing score using the Landry index and the asepsis score was measured using the criterion asepsis point score and again the wound was covered with membrane followed by placement of self-adhesive bandage. The self-adhesive bandage was removed on third day. Wound Healing score and asepsis score were measured on the third, and seventh day, and after two weeks for both the groups.

Data analysis 

The data were collected using the Kobotool kit. It was statistically analyzed using SPSS software version 25.0. Mann-Whitney U test (IBM Corp., Armonk, NY) was performed for intergroup comparison. A p-value < 0.05 was considered to be significant.

## Results

**Figure 1:**
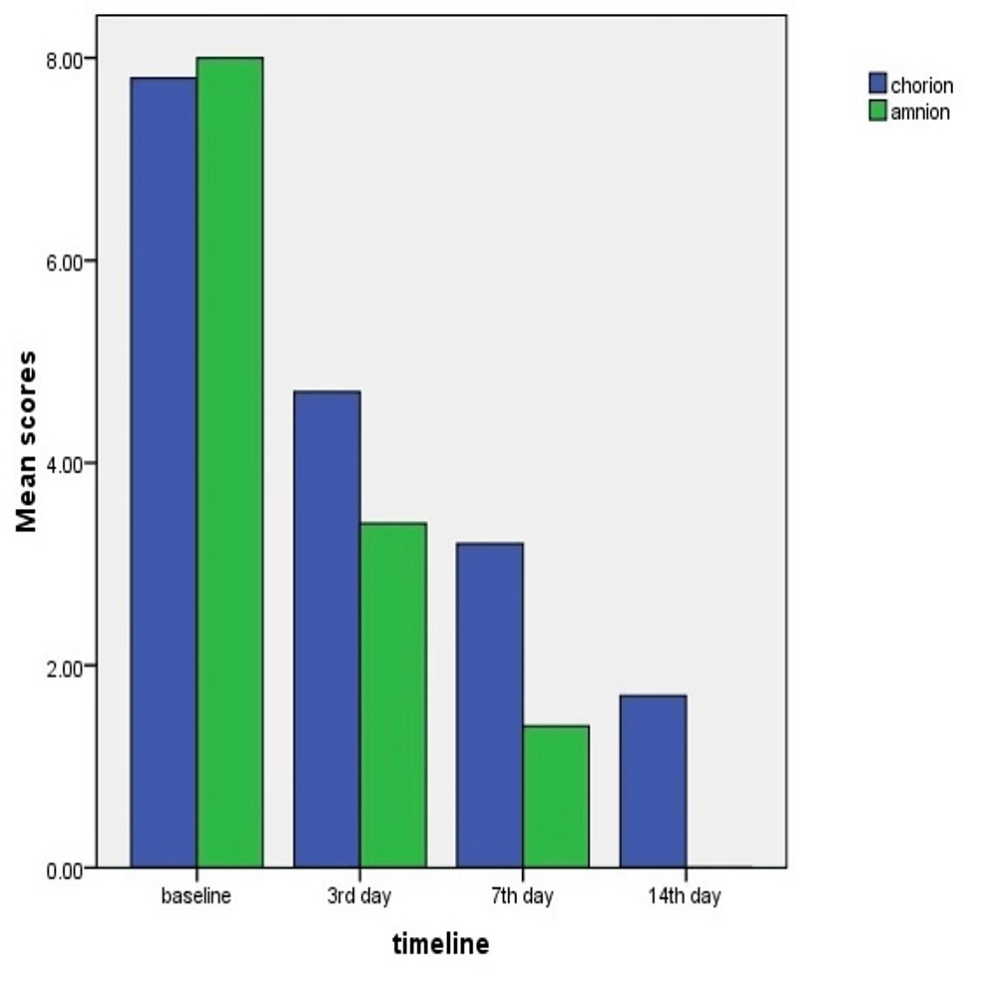
Bar graph comparing the healing period between group1 (chorionic membrane) and group 2 (amniotic fluid)

A total of 20 subjects were screened and enrolled in the study for the two-week run-in period. At the study enrolment, statistically significant differences were observed in patient characteristics, wound size, or wound duration between the study groups. The wound area presents unhealed on the first day, third day, seventh day, and 14th day of amnion and chorion membranes are presented in Figure 1, which shows patients treated with amnion membrane show a gradual decrease in wound size and the wound got completely healed by second week whereas the wound remained unhealed by second week to those treated with chorion membrane. Table 1 shows the mean and standard deviation of the asepsis scores of the two groups.

**Table 1:** Mean and standard deviation of asepsis scores of the two groups

Time interval	Group 1 (chorion)		Group 2 (amnion)	
	Mean	Standard Deviation	Mean	Standard Deviation
Baseline	7.80	1.476	8.00	1.633
3^rd^ day	4.70	0.483	3.40	0.516
7^th^ day	3.200	0.632	1.40	0.516
14^th^ day	1.70	0.483	0.00	0.000

**Figure 2:**
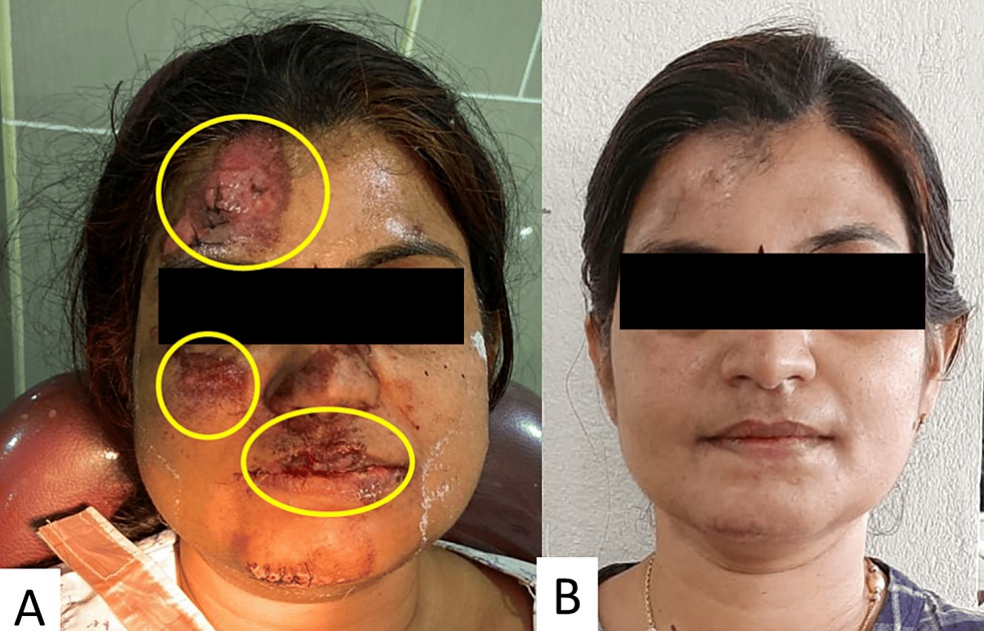
(A) Before and (B) after results of chorionic membrane treatment in a 32-year-old female *Patient consent was taken before using the images as a reference for the healing results.

Figures 2A, 2B show the before and after results of the chorionic membrane treatment in a 32-year-old female. The regions of importance in the application of the membrane have been marked.

## Discussion

For over 100 years, amniotic grafts have been used for a range of medical procedures which demonstrated improved wound healing. Although patients treated with placental-derived grafts showed enhanced recovery, the precise causes of this improved healing were unknown in these earlier investigations. Previous studies have established that advanced wound therapies promoted wound closure, resulting in more compatible and rapid complete healing [10]. Several studies reported the use of amnion in various organ reconstruction procedures, as well as the treatment of wounds of various types. In 1910, Davis [1] was the first person to demonstrate the surgical use of an amnion membrane. Since then, amnion membrane wound healing effectiveness in ocular, diabetic, neurovascular, venous stasis ulcers and various post-surgical and traumatic wounds has been reported. The chorion is the outer layer of the mother cell. Chorion when used alone was not very effective in regenerative medicine, hence the combination of amnion and chorion has become very popular. The thicker chorion accounts for about 75% of the growth factors present when they are utilized together with an amnion membrane. The present study was done to elucidate the efficiency of both amnion and chorion in facial wound healing [4-6].

In the present study patients treated with amnion showed a gradual decrease in wound size and also the wound completely healed within a two-week time period when compared with those treated with chorion the wound remained unhealed by a two-week time period. No pain, inflammation, or scarring were produced in either group. Consistent with the study demonstrated by Schmidt et al., the amnion is made up of three types of material: structural collagen and extracellular matrix biologically active cells and regenerative molecules [23]. A similar study by Cornwell et al. stated that amnion has the property of cellular migration and proliferation [11]. Arai et al. stated that amnion is durable and puncture-resistant [24]. Sham and Sultana stated that amnion is elastic and translucent [25]. Tseng et al. stated that amnion undergoes axonal regeneration [12] showing similar trends as seen in this study.

Schmidt et al. stated that the amnion membrane appears to be safe in its overall use and plays a role significantly in the regeneration of various soft tissues [25]. Chen et al. stated that it is cost-effective in wound care [25-28]. A minimal dressing change is required and with a low need for application meets these requirements. Zelen et al. stated that the dehydrated human amnion/chorion membrane is operationally efficient so that it can be stored at room temperature for about five years [13].

The exact mechanism by which amnion-induced healing was largely unknown. It has been demonstrated in the literature that accelerated keratinocyte migrates from the wound edge and induces differentiation, which thereby produces intact epithelium. JNK regulator of c-jun was reported to be the important factor for the epithelization simulated by the amniotic member at the wound healing borders [29].

Limitations of this study are that this study was carried out only in minimal groups. The observation can be done using histopathological analysis in future studies to provide reliable results. The present study led to the conclusion that while comparing the efficacy of the amnion and the chorion membrane, the amnion membrane has a quicker healing effect when compared with the chorion membrane. More research and clinical studies were required to completely explain their enormous potential which will provide a better way in the field of Oral and maxillofacial surgery.

## Conclusions

Dehydrated human amnion/chorion membranes play an important role in many fields because of their therapeutic healing effect. Expanding the knowledge to the field of Oral and Maxillofacial Surgery and comparing their efficacy, Patients treated with amnion membrane showed a decrease in wound size, and the wound got completely healed by second week whereas the wound remained unhealed by second week to those treated with chorion membrane. No pain, inflammation, or scarring was produced in either group. So, the amnion membrane heals wounds quicker than the chorion membrane.

## References

[1] Pham-Dang N, Barthélémy I, Orliaguet T, Artola A, Mondié JM, Dallel R (2024). Etiology, distribution, treatment modalities and complications of maxillofacial fractures. Med Oral Patol Oral Cir Bucal.

[2] Bochlogyros PN (1985). A retrospective study of 1,521 mandibular fractures. J Oral Maxillofac Surg.

[3] Jeschke MG, van Baar ME, Choudhry MA (2020). Burn injury. Nat Rev Dis Primers.

[4] Gushiken LF, Beserra FP, Bastos JK, Jackson CJ, Pellizzon CH (2021). Cutaneous wound healing: an update from physiopathology to current therapies. Life (Basel).

[5] Chhabra S, Chhabra N, Kaur A, Gupta N (2017). Wound healing concepts in clinical practice of OMFS. J Maxillofac Oral Surg.

[6] Gilliver SC, Ashworth JJ, Ashcroft GS (2007). The hormonal regulation of cutaneous wound healing. Clin Dermatol.

[7] Schultz GS, Chin GA, Moldawer L (2011). Principles of wound healing. Mechanisms of Vascular Disease: A Reference Book for Vascular Specialists.

[8] Wu Y, Chen L, Scott PG, Tredget EE (2007). Mesenchymal stem cells enhance wound healing through differentiation and angiogenesis. Stem Cells.

[9] (1998). Grafts made from amniotic membrane; methods of separating. https://www.ncbi.nlm.nih.gov/books/NBK567771/.

[10] Solomon A, Espana EM, Tseng SCG (2003). Amniotic membrane transplantation for reconstruction of the conjunctival fornices. Ophthalmology.

[11] Solomon A, Rosenblatt M, Monroy D, Ji Z, Pflugfelder SC, Tseng SC (2001). Suppression of interleukin 1alpha and interleukin 1beta in human limbal epithelial cells cultured on the amniotic membrane stromal matrix. Br J Ophthalmol.

[12] Koob TJ, Rennert R, Zabek N (2013). Biological properties of dehydrated human amnion/chorion composite graft: implications for chronic wound healing. Int Wound J.

[13] Zelen CM, Serena TE, Denoziere G, Fetterolf DE (2013). A prospective randomised comparative parallel study of amniotic membrane wound graft in the management of diabetic foot ulcers. Int Wound J.

[14] Akle CA, Adinolfi M, Welsh KI (1981). Immunogenicity of human amniotic epithelial cells after transplantation into volunteers. Lancet.

[15] Lyons AB, Chipps LK, Moy RL, Herrmann JL (2018). Dehydrated human amnion/chorion membrane allograft as an aid for wound healing in patients with full-thickness scalp defects after Mohs micrographic surgery. JAAD Case Rep.

[16] Laurent I, Astère M, Wang KR, Cheng QF, Li QF (2017). Efficacy and time sensitivity of amniotic membrane treatment in patients with diabetic foot ulcers: a systematic review and meta-analysis. Diabetes Ther.

[17] Hao Y, Ma DH, Hwang DG (2000). Identification of antiangiogenic and antiinflammatory proteins in human amniotic membrane. Cornea.

[18] King AE, Paltoo A, Kelly RW, Sallenave JM, Bocking AD, Challis JR (2007). Expression of natural antimicrobials by human placenta and fetal membranes. Placenta.

[19] Talmi YP, Sigler L, Inge E (1991). Antibacterial properties of human amniotic membranes. Placenta.

[20] Gupta A, Kedige SD, Jain K (2015). Amnion and chorion membranes: potential stem cell reservoir with wide applications in periodontics. Int J Biomater.

[21] Le Q, Deng SX (2019). The application of human amniotic membrane in the surgical management of limbal stem cell deficiency. Ocul Surf.

[22] Hao Y, Ma DH, Hwang DG, Kim WS, Zhang F (2000). Identification of antiangiogenic and antiinflammatory proteins in human amniotic membrane. Cornea.

[23] Kothiwale S, Ajbani J (2018). Evaluation of anti-inflammatory effect of chorion membrane in periodontal pocket therapy: a clinical and biochemical study. J Indian Soc Periodontol.

[24] Veves A, Falanga V, Armstrong DG, Sabolinski ML (2001). Graftskin, a human skin equivalent, is effective in the management of noninfected neuropathic diabetic foot ulcers: a prospective randomized multicenter clinical trial. Diabetes Care.

[25] Schmidt W (1992). The amniotic fluid compartment: the fetal habitat. Adv Anat Embryol Cell Biol.

[26] Arai N, Tsuno H, Okabe M, Yoshida T, Koike C, Noguchi M, Nikaido T (2012). Clinical application of a hyperdry amniotic membrane on surgical defects of the oral mucosa. J Oral Maxillofac Surg.

[27] Sham Sham, Ehtaih & Sultana, Nishant Nishant (2011). Biological wound dressing -role of amniotic membrane. https://www.researchgate.net/publication/265159197_Biological_wound_dressing_-role_of_amniotic_membrane.

[28] Huebner EA, Strittmatter SM (2009). Axon regeneration in the peripheral and central nervous systems. Results Probl Cell Differ.

[29] Zelen CM, Serena TE, Fetterolf DE (2014). Dehydrated human amnion/chorion membrane allografts in patients with chronic diabetic foot ulcers: a long-term follow-up study. Wound Med.

